# Anesthetic practice during cardiac implantable electronic device implant procedures: A retrospective, single-center study

**DOI:** 10.1016/j.ijcha.2023.101312

**Published:** 2023-11-24

**Authors:** Cecilia Veraar, Kamen Dimitrov, Sabine Kappel, Sophie J. Wuthe, Clarence J. Veraar, Arabella Fischer, Isabella Worf, Mohamed Mouhieddine, Luana Mandroiu, Bernhard Moser, N. Patrick Mayr, Cesar Khazen, Edda Tschernko, Michael J. Hiesmayr

**Affiliations:** a Department of Anesthesiology, Intensive Care Medicine and Pain Medicine, Division of Cardiac Thoracic Vascular Anesthesia and Intensive Care Medicine, Medical University of Vienna, 1090 Vienna, Austria; b Department of Cardiac Surgery, Medical University of Vienna, Vienna, Austria; c Center for Medical Statistics, Institute for Medical Data Science, Medical University Vienna, 1090 Vienna, Austria; d Department of Anesthesiology, Intensive Care Medicine and Pain Medicine, Medical University of Vienna, 1090 Vienna, Austria; e Department of Thoracic Surgery, Medical University of Vienna, Vienna, Austria; f Institute of Anaesthesiology, German Heart Centre Munich, Faculty of Medicine, Technical University of Munich, Munich, Germany

**Keywords:** Cardiac implantable electronic device, Pacemakers, Implantable cardioverter defibrillators, Anesthetic intervention, Human resources, Anesthetic standby

## Abstract

**Objectives:**

Data on anesthetic proceedings during cardiac implantable electronic device (CIED) implant procedures are scarce and it remains unclear whether anesthetic care is still required in selected patients.

**Methods:**

In this retrospective, single center study we assessed the prevalence of intraoperative anesthetic management comprising anesthetic standby, sedation or general anesthesia as well as anesthetic and procedural complications. We analyzed pre-existing and perioperative risk factors related to procedure-related adverse outcome such as perioperative cardiopulmonary resuscitation (CPR) and 30-day mortality in a uni- and multivariable analysis.

**Results:**

In total, PM and ICD insertion were performed in up to 85% and 58% under anesthetic standby, with an increasing tendency over time.

Overall, Cardiopulmonary resuscitation (CPR) was required in 59 patients. Acute heart failure (AHF) was the only independent pre-existing risk factor for CPR and for 30-day mortality. Sedation and general anesthesia had a significantly increased odds ratio for CPR compared to anesthetic standby. The risk for CPR significantly decreased during the study period.

**Conclusions:**

Over the years anesthetic practice during CIED implant procedures shifted from mixed anesthetic proceedings to mainly standby duties. The prevalence of complications and emergency measures is low, however not uncommon. Accordingly, the presence of an anesthesiologist should be further guaranteed when sedatives were titrated and in AHF patients. However, in patients receiving local anesthetic infiltration only, it seems safe to perform CIED implant procedures without anesthetic standby.

## Graphical abstract

**Figure:**
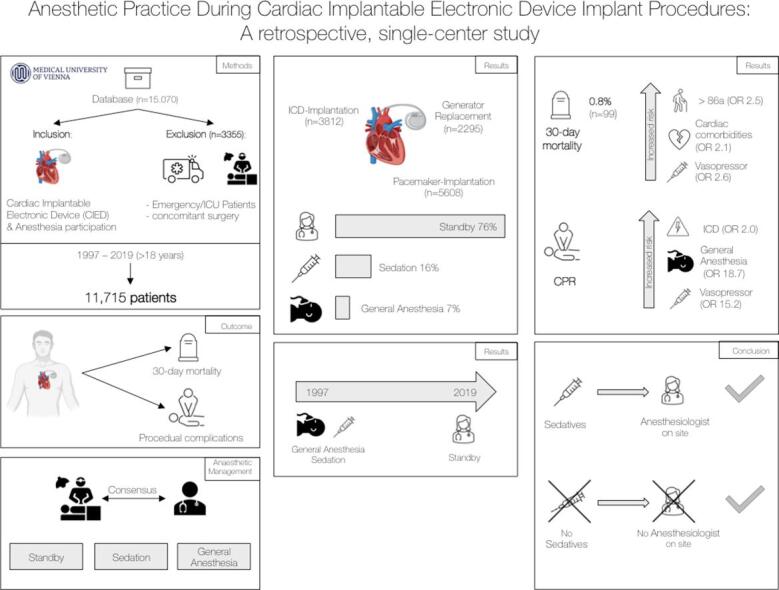

## 1 Introduction

On October 8th, 1958, the world's first fully implantable pacemaker was inserted through thoracotomy and in general anesthesia [1], [2], [3], [4], marking the beginning of the era of cardiac implantable electronic device (CIED) therapy. Technological developments and growing experience have revolutionized implantation techniques since then, enabling less invasive CIED placements with a minimal perioperative risk independent of the applied anesthetic intervention [5].

Even though, general anesthesia for CIED insertion is safe, it is usually no longer required for the implantation of most of the devices, such as pacemakers (PM), implantable cardioverter defibrillators (ICD) and generator replacements [6], [7], [8]. Rather, local anesthetic infiltration of the surgical field is generally used for CIED insertion [9], [10]. The role of anesthesiologists therefore shrank to a minimal role performing mainly standby duties or sedations during local anesthesia in this vulnerable patients’ cohort [11]. However, some patients require sedation or general anesthesia, according to pre-existing factors such as a history of anxiety or difficulties tolerating the procedure. In other patients anesthetic standby has to be extended to sedation or even general anesthesia, due to severe procedural complications occurring in up to 1–3% of all operations [12]. At our institution, anesthesiologists remain always present in the operating room for all CIED procedures to be immediately available to offset emergency measures. However, all over the world, anesthetic management for CIED insertion vary greatly among different institutions ranging from nurses being present in the operating room to anesthetic standby, sedation or even general anesthesia [5], [13].

In addition, in light of the imminent shortage of specialists and nurses and in the presence of anesthetic and procedural technological advances in the field of CIED procedures it becomes crucial to safe extended anesthetic interventions requiring anesthesiological staff for selected patients with pre-existing or perioperative risk factors for adverse outcome [14], [15].

Data on anesthetic proceedings during cardiac implantable electronic device (CIED) implant procedures are scarce and it remains unclear whether anesthetic care is still required in selected patients.

Therefore, the main objective of this study was to define pre-existing and perioperative risk factors for adverse outcome after CIED insertion and to determine vulnerable patients who may benefit from anesthetic standby.

Thus, we analyzed our institutional experience of anesthetic proceedings and quality of care in the case of routine PM and ICD insertion and generator replacement in a minor procedure room over more than 20 years. We assessed the prevalence of intraoperative anesthetic management comprising anesthetic standby, sedation or general anesthesia as well as anesthetic and procedural complications. We assessed pre-existing and perioperative risk factors related to procedure-related adverse outcome such as perioperative cardiopulmonary resuscitation (CPR) and 30-day mortality in a uni- and multivariable analysis.

## 2 Materials and Methods

### 2.1 Ethical approval

The study was conducted in accordance with the Declaration of Helsinki (as revised in 2013) and was approved by the Ethics Committee of the Medical University of Vienna (EK1853/2022). The data collection was performed in accordance with the approved ethical guidelines.

### 2.2 Study design and patients selection

This work was designed as a retrospective, descriptive single-center cohort study. We included 15,070 consecutive American Society of Anesthesiologists III and IV patients who underwent a CIED implant procedure between 1997 and 2019 in a designated procedure room, at the Medical University of Vienna. The minor procedure room is an operating room dedicated to smaller and ambulatory interventions. We excluded all patients who underwent a concomitant surgical procedure in addition to CIED implantation, intensive care and emergency patients who underwent CIED insertion in the general operating room and patients who were younger than 18 years. Study endpoints included the prevalence for intraoperative anesthetic interventions, the prevalence of anesthetic and procedural complications, and predictors of a poor outcome comprising cardiopulmonary resuscitation (CPR) and 30-day mortality. Data was extracted from a patient data management tool introduced at our institution in 1997. Data on 24 h and 30 day (30d) mortality was obtained from Statistics Austria via the Medical University of Vienna.

### 2.3 Anesthetic management

All electively enrolled patients received standard perioperative monitoring after entering the dedicated procedure room. Defibrillator paddles were placed as appropriate. Patients undergoing CIED implant procedures under anesthetic standby received either a premedication with midazolam or no medication. The cardiac surgeon infiltrated local anesthetics into the surgical field. Patients with anxiety or pain received additional sedation using Propofol only, Propofol and Midazolam or Propofol and Opioids. General anesthesia was induced referring to our institutional standards. Accordingly, we categorized anesthetic interventions into one of the following categories: a) anesthetic standby b) sedation or c) general anesthesia as detailed in Table 1. According to our institutional standards an anesthesiologist was always present in the procedure room during CIED implant procedures independently of the selected anesthetic procedure. The decision on anesthetic proceedings for CIED implant procedures was taken in accordance with the cardiac surgeon after interdisciplinary discussion of all relevant patient data.

**Table 1:** Categorizations of anesthetic interventions during CIED procedures are listed below.

	**Anesthetic interventions**		
	**Standby**	**Sedation**	**General anesthesia**
**Infiltration**	Local Anesthesia	Local Anesthesia	With/without Local Anesthesia
**Anesthetics**	No premedication OR Premedication with Midazolam	Propofol and Midazolam OR Propofol and Fentanyl OR Propofol and Morphine OR Propofol and Remifentanil	Sevoflurane AND/OR Propofol AND fentanyl OR Remifentanil WITH/WITHOUT Rocuronium or Cisatracurium
**Airway**	Oxygen mask	Oxygen mask	Intubation, Laryngeal mask
**Anesthesiologist**	Present	Present	Present

*Anesthetic Standby* consists of local anesthesia, carried out by the surgeon in addition to premedication, or no premedication and the use of an oxygen mask. *Sedation* comprises local anesthesia, carried out by the surgeon in addition to listed anesthetics and the use of an oxygen mask. *General anesthesia* with/without local anesthesia includes total intravenous anesthesia or balanced anesthesia, with/without muscle relaxation and airway protection.

### 2.4 Statistical analyses

Demographic and clinical data were presented using descriptive statistics. The Shapiro–Wilk test was used to examine whether variables were normally distributed. Mean ± standard deviation (SD) and median and interquartile range (25% percentile, 75% percentile) were given for continuous variables. Categorical variables were shown as frequency (percentage). Further, we performed univariable and multivariable logistic regression analysis to determine independent factors associated with poor outcome comprising cardiopulmonary resuscitation (CPR) and 30-day mortality. Odds ratios (OR) were calculated for basic demographic data such as age and gender, underlying diagnoses, procedures and anesthetic interventions and complications.

Statistical analyses were performed using the IBM SPSS statistics 27.0 (SPSS, Inc., Armonk, NY, USA) software. Figures were performed using Graph Pad Prism Version 9 (GraphPad Software, Inc., BOSTON, MA, USA) software.

## 3 Results

**Fig. 1:**
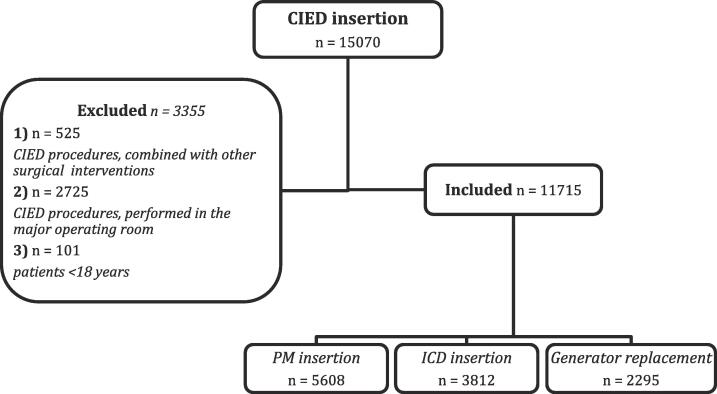
**CONSORT Flowchart of CIED Implant Procedures: Enrollment, Exclusion, and Final Cohort Selection.** The flowchart shows the number of CIED implant procedures performed during the study period. The final cohort consists of consecutive cases of CIED implant procedures that were conducted in adult patients within a dedicated procedure room at our center during the study period. Cases performed outside of the dedicated procedure room, patients under 18 years of age, and cases where a concomitant surgical procedure was performed were excluded from the final analysis. *ICD* implantable cardioverter-defibrillator*, ICU* intensive care unit, *PM* pacemaker.

This study included 15,070 consecutive patients who underwent a CIED implant procedure between 1997 and 2019 in a designated procedure room at our institution. We excluded 525 patients who underwent a concomitant surgical procedure at the time of the CIED implant procedure, 2795 intensive care or emergency patients who underwent CIED implantation in the general operating room and 101 patients who were younger than 18 years as detailed in Fig. 1.

Patients' demographic and clinical data are listed in Table 2**.** In total, 71.3 % (n = 10744) of all included patients underwent CIED insertion due to arrhythmia, 34.9 % (n = 5259) had an atrioventricular (AV) block. Pre-existing comorbidities, comprising cardiomyopathies and coronary heart diseases were present in 9.3% (n = 462) and 3.9% (n = 462), respectively. In total, 3.7% (n = 428) underwent Cardiopulmonary resuscitation (CPR), 2.1% (n = 249) had a ventricular fibrillation, 20.3 % (n = 2376) had a myocardial infarction and 10.8 (n = 1267) patients had a syncope prior CIED insertion. Non-cardiac comorbidities such as diabetes, COPD and stroke was reported in 17.1% (n = 2000), 8.9% (n = 1048) and 6.1 (n = 720) patients.

**Table 2:** Patient characteristics and implant data.

	**PM Insertion**	**ICD Insertion**	**Generator** **Replacement**
**Age** mean ± SD	71 ± 12	62 ± 13	72 ± 15
**Female** n (%)	2186 (39.0)	836 (21.9)	932 (40.6)
**BMI** kg/m^2^ mean ± SD	25 ± 4	26 ± 4	26 ± 5
**Diagnosis**			
**AV block** n (%)	2453 (43.7)	731 (19.2)	900 (39.2)
**Arrhythmia** n (%)	3849 (68.6)	2945 (77.3)	1561 (68.0)
**Pre-existing Comorbidities**			
**CMP** n (%)	209 (3.7)	782 (20.5)	95 (4.1)
**CHD** n (%)	137 (2.4)	263 (6.9)	62 (2.7)
**CPR** n (%)	58 (1.0)	256 (9.8)	28 (1.2)
**VF (recent)** n (%)	13 (0.2)	231 (6.1)	5 (0.2)
**AHF** n (%)	408 (7.3)	376 (9.9)	64 (2.8)
**MCI** n (%)	802 (14.3)	1333 (35)	241 (10.5)
**Syncope** n (%)	821 (14.6)	331 (8.7)	115 (5.0)
**Diabetes** n (%)	941 (16.8)	682 (17.9)	377 (16.4)
**COPD** n (%)	475 (8.5)	403 (10.6)	170 (7.4)
**Stroke** n (%)	366 (6.5)	204 (5.4)	150 (6.5)

Patients who underwent CPR prior to CIED insertion were listed under “CPR”. Patients who underwent CIED insertion due to ventricular fibrillation were listed under “VF”. Patients with insulin- and insulin independent diabetes were given under “diabetes”.

*AHF* acute heart failure, *AV* atrioventricular, *BMI* body mass index, *CHD* Coronary heart disease*, CMP* cardiomyopathy, *COPD* chronic obstructive pulmonary disease, *CPR* cardiopulmonary resuscitation, *MCI* myocardial infarction, *n* number, *SD* standard deviation, *VF* ventricular fibrillation,

### 3.1 Anesthetic interventions: Trends and patterns

**Fig. 2:**
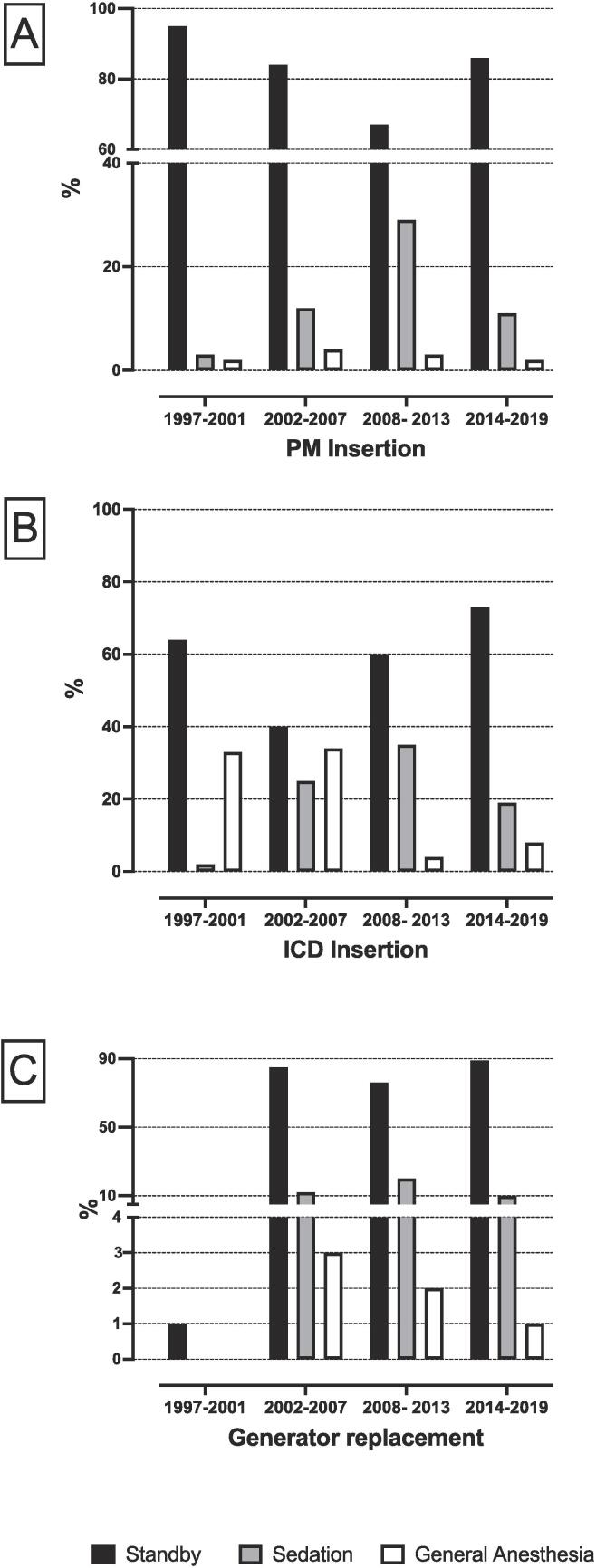
**Applied anesthetic interventions for different CIED implant procedures over four time periods are detailed below.** The percentage of patients undergoing PM insertion (**A**), ICD insertion (**B**) and generator replacement (**C**) over 4 time periods are shown above. *ICD* implantable cardioverter-defibrillator *PM pacemaker.*

A summary of the utilization of different types of anesthetic interventions for different types of CIED implant procedures is shown in Fig. 2A, B and C and the supplementary material Table 1**.**

Overall, anesthetic standby was the most frequently used anesthetic intervention, applied in 76% (n = 8942) of all procedures. PM and generator replacement procedures were mainly performed under anesthetic standby, ranging from 83.9 to 95.6% and 76.4–100%. The use of anesthetic standby increased for ICD implant procedures over the years, ranging from 40% to 73%.

Sedation was the second most commonly used anesthetic intervention with an overall use of 16% (n = 1869) among all CIED implant procedures. There was anincreased use of sedation throughout the first three consecutive implant eras followed by a slight decrease in the final implant era applying for all types CIED implant procedures.

General anesthesia was the least frequently used anesthetic intervention for all procedures, applied in 7% (n = 904) of all patients. It was mainly used for ICD insertion at the beginning of the CIED implant era, but steadily decreasing over time, from 34.1% as a maximum to 4.2% as a minimum.

For PM insertion and generator replacement procedures the use of general anesthesia was low with no considerable fluctuations throughout the implant eras, ranging from 1.7 to 2.0% and 1.4–3.4%.

### 3.2 Emergency measures and anesthetic and procedure-related complications according to CIED implant procedures and anesthetic interventions

Emergency measures and anesthetic and procedure-related complications according to CIED implant procedures and anesthetic interventions are detailed in the supplementary material Table 2 and 3**.**

Over 20 years 1586 emergency measures were reported. In total 3.8 % (n = 446) of all patients underwent defibrillation or cardioversion; the highest prevalence was reported for ICD implants and under general anesthesia. However, only 0.39 % (n = 59) of all patients required CPR.

In our study period anesthetic complications occurred 59 times. Hypoxemia mainly occurred during PM and ICD implantation procedures and under general anesthesia.

### 3.3 Predictors for cardiopulmonary resuscitation and 30-day mortality

Predictors for CPR and 30-day mortality were detailed in Table 3 A and B. Older patients were significantly less likely to require CPR compared to younger patients ≤ 55 years. Compared to the early years from 1997 to 2001 the OR decreased significantly over time, with a decreased OR for 2002–2007, 2008–2013 and 2014–2019 in the multivariable model. Regarding comorbidities, AHF was the independent risk factor for CPR in the multivariable model.

Compared to PM insertion, ICD insertion had a significantly increased OR for CPR [OR = 2.0 (95%CI: 1.1–3.4); p = 0.008] and generator replacement had a significantly lower OR [OR = 0.2 (95%CI: 0.0–0.8); p = 0.030] in the univariable, but not multivariable model. In contrast, general anesthesia had a significantly increased association with CPR compared to standby anesthesia [OR = 18.7 (95%CI: 10.5–33.4); p= <0.001]. However, in the multivariable model sedation had the highest OR for CPR. Furthermore, vasopressor administration and defibrillation/cardioversion had a significantly increased association for CPR in the multivariable model. External pacing revealed no significant association with CPR. Hypoxemia was the only anesthetic complications with an independent risk for CPR (Table 3A).

**Table 3:** logistic regression for CPR and 30d mortality Logistic regression was performed to analyze the association of CPR (**A**) 30-day mortality (**B**) for demographic data, over the years, diagnoses, procedures, anesthetic interventions and complications. The reference value was marked with an arterix. Significant values were marked in bold.

**Univariable logistic regression**				**Multivariable logistic regression**		
**CPR**	**OR**	**CI 95%**	**p-value**	**OR**	**CI 95%**	**p-value**
***Gender***						
male*	1.0					
female	1.0	0.6–1.8	0.764			
***Age (years)***						
<=55*	1.0			1.0		
56–65	0.6	0.3–1.3	0.262	0.9	0.4–2.1	0.881
66–75	0.6	0.3–1.3	0.241	1.2	0.5–2.5	0.629
76–85	**0.3**	0.1–0.6	**0.004**	0.5	0.2–1.5	0.299
>=86	0.6	0.2–1.6	0.340	2.8	0.9–8.6	0.071
***Over the years***						
1997–2001*	1.0			1.0		
2002–2007	**0.5**	0.2–0.8	**0.019**	**0.1**	0.0–0.2	**<0.001**
2008–2013	**0.2**	0.09–0.5	**<0.001**	**0.1**	0.0–0.4	**<0.001**
2014–2019	**0.1**	0.05–0.4	**<0.001**	**0.1**	0.0–0.4	**0.01**
**Diagnoses**						
CHD (yes)	2.2	0.9–5.6	0.081			
CMP (yes)	1.1	0.4–2.5	0.811			
AV Block (yes)	1.1	0.7–2.0	0.506			
Arrhythmia (yes)	1.5	0.8–2.9	0.159			
CPR (yes)	1.4	0.4–4.5	0.558			
VF (yes)	0.7	0.1–5.7	0.813			
AHF (yes)	**3.3**	1.7–6.2	**<0.001**	**2.1**	**1.1**–**4.9**	**0.037**
MCI (yes)	1.5	0.9–2.8	0.108			
Syncope (yes)	1.2	0.6–2.7	0.497			
Diabetes (yes)	1.5	0.8–2.7	0.176			
COPD (yes)	1.1	0.4–2.6	0.741			
Stroke (yes)	1.7	0.7–0.4	0.203			
***Procedure***						
PM*	1.0			1.0		
ICD	**2.0**	1.1–3.4	**0.008**	0.9	0.4–1.8	0.905
Generator replacement	**0.2**	0.04–0.8	**0.030**	0.6	0.1–2.9	0.643
**Anaesthetic actions**						
Standby*	1.0			1.0		
Sedoanalgesia	2.1	0.9–4.9	0.075	**5.0**	2.3–10.7	**<0.001**
General anaesthesia	**18.7**	10.5–33.4	**<0.001**	2.3	0.8–6.3	0.090
Vasopressor (yes)	**15.2**	8.8–26.2	**<0.001**	**10.8**	4.9–23.8	**<0.001**
External pacing (yes)	1.9	0.8–4.5	0.119			
Defibrillation/Cardiov. (yes)	**24.2**	14.4–40.8	**<0.001**	**10.1**	5.1–19.9	**<0.001**
**Complications**						
Aspiration (yes)	0.0	0.0	0.999			
Laryngospasm (yes)	0.0	0.0	0.999			
Hypoxemia (yes)	**36.7**	12.3–109.8	**<0.001**	**7.5**	1.8–34.5	**0.006**
Intubation difficulty (yes)	**104.0**	25.3–426.2	**<0.001**	7.1	0.5–92.0	0.131

**Univariable logistic regression**				**Multivariable logistic regression**		
**30d mortality**	**OR**	**CI 95%**	**p-value**	**OR**	**CI 95%**	**p-value**
***Gender***						
male*	1.0					
female	0.7	0.4–1.1	0.194			
***age (years)***						
<=55*	1.0			1.0		
56–65	1.1	0.5–2.4	0.798	1.0	0.4–2.3	0.869
66–75	0.8	0.3–1.7	0.592	0.8	0.3–1.8	0.644
76–85	1.8	0.9–3.6	0.067	1.9	0.9–3.9	0.061
>=86	**2.5**	0.1–5.5	**0.020**	**2.9**	1.3–6.8	**0.010**
***Over the years***						
1997–2001*	1.0					
2002–2007	1.0	0.5–1.7	0.914			
2008–2013	1.3	0.7–2.2	0.306			
2014–2019	0.5	0.2–1.1	0.106			
**Diagnosis**						
CHD (yes)	**2.1**	1.0–4.5	**0.036**	1.7	0–8-3.8	0.128
CMP (yes)	**1.7**	1.0–3.0	**0.041**	1.6	0.9–3.0	0.084
AV Block (yes)	1.1	0.7–1.7	0.546			
Arrhythmia (yes)	1.0	0.6–1.6	0.804			
CPR (yes)	**2.3**	1.14.9	**0.020**	2**.4**	1.0–5.6	**0.039**
VF (yes)	**2.5**	1.0–6.2	**0.048**	1.8	0.6–5.5	0.242
AHF (yes)	**2.7**	1.6–4.6	**<0.001**	**2.3**	1.3–4.0	**0.002**
MCI (yes)	2.1	0.7–1.9	0.431			
Syncope (yes)	**1.8**	1.1–3-1	**0.017**	**1.8**	1.0–3.1	**0.002**
Diabetes (yes)	1.6	0.1–15.5	0.676			
COPD (yes)	**10.1**	1.4–72.4	**0.020**	1.6	0.9–2.8	0.09
Stroke (yes)	**1.3**	0.6–2.8	0.406			
***Procedure***						
PM*	1.0					
ICD	0.6	0.4–1.0	0.103			
Generator replacement	**0.5**	0.2–0.9	**0.049**			
**Anaesthetic actions**						
Standby*	1.0					
Sedoanalgesia	0.6	0.5–2.2	0.706			
General anaesthesia	1.1	0.3–1.2	0.220			
Vasopressor (yes)	**2.6**	1.3–5.2	**0.006**	2.0	0.9–4.4	0.053
External pacing (yes)	0.7	0.2–1.9	0.543			
Defibrillation/Cardiov. (yes)	1.0	0.3–2.9	0.887			
**Complications**						
Aspiration (yes)	0.0	0.0	0.999			
Laryngospasm (yes)	0.0	0.0	0.999			
Hypoxemia (yes)	**9.6**	2.2–41.3	**0.002**	**5.2**	1.1–24.6	**0.036**
Intubation difficulty (yes)	0.0	0.0	0.999			

Hypoxemia was reported as a decrease in arterial oxygen saturation below < 90%.

*AHF* acute heart failure, *AV* atrioventricular, *BMI* body mass index, *CHD* Coronary heart disease*, CI* confidence interval, *CMP* cardiomyopathy, *COPD* chronic obstructive pulmonary disease, *CPR* cardiopulmonary resuscitation, *ICD* implantable cardioverter-defibrillator*, ITN* intubation, *MCI* myocardial infarction, *OR* odds ratio, *PM* pacemaker, *VF* ventricular fibrillation.

Patients older than 86 years had an independent risk for 30-day mortality [OR = 2.9 (95%CI: 1.1–5.5); p = 0.010]. Furthermore, patients with cardiac comorbidities such as CHD and CMP had an elevated risk for 30-day mortality [OR = 2.1 (95%CI: 1.0–4.5); p = 0.036] and [OR = 1.7 (95% CI: 1.0–3.0), p = 0.041] in the univariable model only. Patients who underwent CPR prior to CIED insertion, patients with AHF and patients after syncope had an increased risk for 30-day mortality in the multivariable model. In comparison to PM insertion, generator replacement was associated with a significantly decreased risk for 30-day mortality. Additionally, patients with hypoxemia had a higher OR [OR = 5.2 (95%CI: 1.1–24.6); p = 0.036] as shown in Table 3 **B.**

## 4 Discussion

This retrospective study showed that there was minimal anesthetic and procedural risk for patients undergoing CIED insertion. AHF was the only independent pre-existing risk factor for perioperative CPR and for 30d mortality, suggesting continuing preanaesthesiological assessment in conjunction with anesthetic standby for patients diagnosed with AHF. Both sedation and general anesthesia were independent perioperative risk factors for CPR, pointing out that titration of sedatives benefit from the presence of an anesthesiologist. Even though general anesthesia correlated with emergency measures and anesthetic and procedure-related complications in the entire cohort, this association was more pronounced in patients undergoing PM insertion, indicating that general anesthesia became a surrogate for adverse events, especially in those patients. In line with our findings Mayr et al. found that the need for intubation was not related to sedation, but to AHF and procedure-related complications during CIED insertion [16]. Overall, general anesthesia was rarely performed during our observational period. However, at the beginning of the CIED implant era, it was more often applied for ICD insertion, since ICD systems were tested after implantation by inducing arrhythmia. For PM insertion and generator replacement procedures the use of general anesthesia was low with no considerable fluctuations over time. Our findings emphasize that general anesthesia lost its role as a standard anesthetic intervention during CIED procedures. Moreover, the risk of CPR decreased significantly over the years pointing out an institutional learning curve in conjunction with technical advances in the field of CIED insertion.

In total, the CPR rate of our cohort was at 0.39%. In literature, we found a pre-incision cardiac arrest rate of 0.18% for patients undergoing cardiac surgery and a perioperative and postoperative CPR rate of 0.03% and 0.33% for patients undergoing non-cardiac surgery [17], [18]. In our study, patients undergoing ICD insertion had an increased risk for CPR compared to patients undergoing PM insertion and generator replacement in the univariable, but not multivariable model. In our entire cohort the 24-hour mortality rate was at 0.02% and the 30-day mortality was at 0.8%. In literature the in-hospital mortality of patients undergoing open-heart surgery predicted by the additive EuroSCORE was 6.24% [19]. For patients undergoing non-cardiac surgery a mortality rate of 0.16% was reported [20]. Our relatively high 30-day mortality however, seems not related to the procedure itself. According to the logistic regression analysis, risk factors for 30-mortality include patients with an increased age, patients with cardiac comorbidities and Intermediate Care patients requiring vasopressors and patients with hypoxemia, independently of the procedure.

In our study, anesthetic standby was the most frequently used anesthetic intervention during CIED implant procedures. PM and generator replacement procedures were mainly performed under anesthetic standby. However, there was a trend towards a decreased use of anesthetic standby in PM implants throughout the successive eras that mirrored the use of sedation. In contrast, anesthetic standby trended towards an increased use in ICD implant procedures over the years, reflecting a decreasing tendency of performing general anesthesia. Nonetheless, in patients requiring a subcutaneous ICD system general anesthesia is still required, as a greater amount of tissue has to be dissected [21]. To over come this limitation of general anesthesia Migliore and colleagues recently confirmed practicability and safety of ultrasound-guided serratus anterior plane block as an alternative anesthetic modality for subcutaneous ICD implantation [22].

At our institution in the past all anesthetic procedures were performed under permanent presence of an anesthesiologist in the procedure room. However, according to the hereby-presented data it’s time to think of performing CIED insertion in selected patients, without AHF, who do not require sedatives in the absence of an anesthesiologist.

Previous studies reported a higher complication rate during minimally invasive interventions without presence of an anesthesiologist, since a dedicated anesthesia team allows immediate management of hemodynamic instability and/or life-threatening procedural complications [23], [24], [25]. Trouve-Buisson et al. reported an adverse event rate of 29% during CIED implant procedures in high-risk patients. However, 92% of all reported interventions were performed under deep sedation, an anesthetic technique that is associated with a high number of respiratory adverse events [26]. Accordingly, procedural protocols must be available for anesthesiologists and other specialized personnel to ensure rapid response to emergency situations if no anesthesia team remains on site [27].

In general, local anesthesia without anesthesiologist on site is associated with lower total hospital costs due to a reduced total anesthesia-controlled operating room time, a reduction in post-anesthesia recovery time and an overall shorter length of stay [14]. According to upcoming resource constraints these financial considerations become more important for decision-making in our daily clinical practice [28].

Our findings suggest that the presence of an anesthesiologist must be further guaranteed when sedatives were titrated and in patients with AHF. In contrast, for patients receiving local anesthetic infiltration with and without premedication, it seems safe to perform CIED implant procedures without anesthetic standby.

This study has several limitations owing to its retrospective design. Therefore, we could only extract complications that were incorporated in to our data management tool. Furthermore, we could not determine whether patients switched from one anesthetic procedure to another. As a result we could only analyze the final anesthetic intervention.

Moreover, our data quality is biased by individual efforts of cardiac anesthesiologists to document correctly. In addition, it was not documented whether transvenous or subcutaneous ICDs and single- or biventricular PMs were implanted. Another draw back of this study concerns the long observational period reflecting a heterogeneous picture of PM and ICD technologies and techniques for insertion. Hence, we categorized our data into four time-periods, emphasizing a learning curve of anesthetic and surgical practices of almost two decades. The strength of this study is that exclusively trained anesthesiologists performed anesthetic proceedings throughout the total observational period. Furthermore, our study includes one of the largest cohorts undergoing CIED implant procedures.

Further prospective studies are warranted to analyze how to prevent adverse events during CIED implant procedures.

## 5 Conclusion

Anesthetic procedures during CIED implant procedures shifted from mixed anesthetic proceedings comprising general anesthesia, sedation and anesthetic standby to mainly standby duties over the years. The prevalence of procedure-related complications and emergency measures is low, however not uncommon. Thirty-day mortality after CIED implant procedures seems rather pertaining patients comorbidities than conflicting with procedural management. Accordingly, our data suggests that the presence of an anesthesiologist must be further guaranteed when sedatives were titrated and in patients with AHF. In patients receiving local anesthetic infiltration with and without premedication, it seems safe to perform CIED implant procedures without anesthetic standby. However, trained anesthesia team must remain always available to respond to emergencies within few minutes to off set complications.

**Funding**

The research received no external funding.

**Author Contributions**

C.V. contributed to conception, design, data analysis and writing. M.H. contributed to conception, design, supervision and critical revision. K.D, A.F, L.M and B.M contributed to writing, design and critical revision. C.K contributed to patients' recruitment, data collection and critical revision. I.W and M.M. performed data collection and data analysis. S.K, C.J.V and S.J.W contributed to design, data recruitment and critical revision. N.P.M, A.L and E.T. performed supervision and critical revision. All authors have read and agreed to the published version of the manuscript.

## Declaration of Competing Interest

The authors declare that they have no known competing financial interests or personal relationships that could have appeared to influence the work reported in this paper.
