# Supplementary material for: Anesthetic practice during cardiac implantable electronic device implant procedures: A retrospective, single-center study
Source: Int J Cardiol Heart Vasc. 2023 Nov 24;49:101312. doi: 10.1016/j.ijcha.2023.101312 (PMC10701355; doi:10.1016/j.ijcha.2023.101312)
Supplement: Supplementary Data 1 [file mmc1.docx]

Supplementary Material

Anesthetic Practice During Cardiac Implantable Electronic Device Implant Procedures: A retrospective, single-center study

**Table 1. Applied anesthetic interventions for different CIED implant procedures over four time periods are detailed below.**

|  | | **1997-2001**  n= 2629 | **2002-2007**  n=3713 | **2008- 2013**  n=2979 | **2014-2019**  n=2394 |
| --- | --- | --- | --- | --- | --- |
| **PM Insertion (**n=5608**)** | | | | | |
| General anesthesia | | 36 (1.7) | 63 (3.6) | 32 (3.3) | 17 (2.2) |
| Sedation | | 55 (2.7) | 222 (12.5) | 287 (29.4) | 91 (11.6) |
| Standby | | 1983 (95.6) | 1490 (83.9) | 658 (67.3) | 674 (86.2) |
| **ICD insertion (**n=3812**)** | | | | | |
| General anesthesia | | 183 (33.0) | 390 (34.1) | 50 (4.2) | 74 (7.9) |
| Sedation | | 12 (2.2) | 286 (25.0) | 410 (34.8) | 174 (18.8) |
| Standby | | 359 (64.8) | 467 (40.9) | 717 (60.9) | 690 (73.6) |
| **Generator replacement (**n=2295**)** | | | | | |
| General anesthesia | - | | 27 (3.4) | 23 (2.8) | 9 (1.3) |
| Sedation | - | | 93 (11.7) | 172 (20.8) | 67 (10.0) |
| Standby | 1 (100) | | 675 (84.9) | 630 (76.4) | 598 (88.7) |

*ICD* implantable cardioverter-defibrillator*, PM* pacemaker, *n* number

**Table 2. Complications related to CIED implant procedures.**

|  | **Total** | **PM Insertion** | **ICD Insertion** | **Generator**  **Replacement** |
| --- | --- | --- | --- | --- |
| **Emergency measures n (%)** | | | | |
| Defibrilltion/Cardioversion | 446 (3.8) | 82 (1.5) | 257 (9.4) | 7 (0.3) |
| CPR | 59 (0.5) | 24 (0.4) | 33 (0.9) | 2 (0.1) |
| Vasopressor administration | 439 (3.8) | 127 (2.3) | 261 (6.9) | 51 (2.2) |
| External pacing | 642 (5.5) | 276 (4.9) | 205 (5.4) | 161 (7.0) |
| **Anesthetic and procedure-related complications n (%)** | | | | |
| Aspiration | 4 (0.0) | 1 (0.0) | 1 (0.0) | 2 (0.0) |
| Laryngospasm | 7 (0.1) | 4 (0.1) | 3 (0.1) | - |
| Hypoxemia (<90%) | 27 (0.2) | 15 (0.3) | 11 (0.3) | 1 (0.0) |
| Intubation difficulty | 9 (0.1) | 7 (0.1) | 2 (0.1) | - |
| Pneumothorax | 12 (0.1) | 7 (0.1) | 4 (0.1) | - |
| **Mortality n (%)** | | | | |
| Mortality (24h) | 3 (0.0) | 3 (0.1) | - | - |
| Mortality (30d) | 98 (0.8) | 58 (1.0) | 27 (0.7) | 13 (0.6) |

*CPR* cardiopulmonary resuscitation, *ICD* implantable cardioverter-defibrillator, *n* number, *PM* pacemaker, *Defibrillation/Cardioversion* was determined when performed by the anesthesiologist. *External pacing* comprises patients who had an external temporary pacemaker in situ.

**Table 3. Complications related anesthetic interventions for CIED implant procedures.**

|  |  | | **General**  **Anesthesia** | **Sedation** | **Anesthetic standby** |
| --- | --- | --- | --- | --- | --- |
| **Emergency measures n (%)** | | | | | |
| Defibrillation/Cardioversion | |  | 215 (23.8) | 84 (4.5) | 147 (1.6) |
| CPR | |  | 33 (3.7) | 8 (0.4) | 18 (0.2) |
| Vasopressor administration | |  | 182 (20.2) | 151 (8.1) | 106 (1.2) |
| External pacing | |  | 19 (2.1) | 49 (2.6) | 574 (6.4) |
| **Anesthetic and procedure-related complications n (%)** | | | | | |
| Aspiration |  | | 1 (0.1) | - | 3 (0.0) |
| Laryngospasm |  | | 4 (0.1) | 3 (0.1) | - |
| Hypoxemia (<90%) |  | | 10 (1.1) | 8 (0.4) | 9 (0.1) |
| Intubation difficulty |  | | 9 (0.9) | - | - |
| Pneumothorax |  | | 3 (0.3) | 2 (0.1) | 7 (0.1) |
| **Mortality n (%)** | | | | | |
| Mortality (24h) |  | | 3 (0.3) | - | - |
| Mortality (30d) |  | | 9 (1.0) | 11 (0.6) | 78 (0.9) |

*CPR* cardiopulmonary resuscitation, *ICD* implantable cardioverter-defibrillator, *n* number, *PM* pacemaker, *Defibrillation/Cardioversion* was determined when performed by the anesthesiologist. *External pacing* comprises patients who had an external temporary pacemaker in situ.

Mortality related to CIED implantation

Thirty-day mortality after CIED implant procedure was 0.8 % (n=99). Of note, three patients (0.02%) died within 24 hours after intervention. The first patient came already moribund with a bronchial carcinoma and brain metastases as a malignant underlying disease. This patient required a pacemaker for bradycardic atrial fibrillation between 20 and 30 beats/min. Insertion and positioning of the ventricular probe was difficult due to an enlarged right ventricle; suddenly there was a myocardial perforation resulting in a cardiac tamponade. Subsequently, the patient was intubated and the tamponade was relieved via subxyphoid pericardiotomy under CPR. During CPR, external or internal pacing failed and CPR did not show an adequate effect; after 25 minutes of unsuccessful CPR, resuscitation measures were stopped. The second patient who died received an uneventful PM insertion for sick sinus syndrome. However, the patient was unresponsive to PM pacing after the operation. Subsequently, the patient was intubated and monitored in the recovery area. The patient died on the following day. According to the necropsy the clinical cause of death was a cardiogenic shock due to a massive perioperative myocardial infarction. The third patient who died had an ischemic cardiomyopathy after a posterior wall infarction and underwent PM insertion for an atrioventricular block III. After probe insertion, ventricular fibrillation suddenly occurred resulting in CPR and defibrillation. After about 45 minutes of CPR, the patient regained its own rhythm, which remained stable only for a short period of time. After 1 hour of unsuccessful CPR, resuscitation measures were discontinued.
